# Nonpolio Enterovirus Activity during the COVID-19 Pandemic, Taiwan, 2020

**DOI:** 10.3201/eid2701.203394

**Published:** 2021-01

**Authors:** Shu-Chen Kuo, Hsiao-Hui Tsou, Hsiao-Yu Wu, Ya-Ting Hsu, Fang-Jing Lee, Shu-Man Shih, Chao A. Hsiung, Wei J. Chen

**Affiliations:** National Health Research Institutes, Zhunan, Taiwan

**Keywords:** COVID-19, coronavirus disease, SARS-CoV-2, severe acute respiratory syndrome coronavirus 2, viruses, respiratory infections, zoonoses, nonpolio enterovirus, nonpharmaceutical, interventions, preventable fraction

## Abstract

In Taiwan, lower nonpolio enterovirus activity during the coronavirus disease pandemic in 2020 compared with 2014–2019 might be attributable to adherence to nonpharmaceutical interventions. The preventable fraction among unexposed persons indicated that 90% of nonpolio enterovirus activity might have been prevented during 2014–2019 by adopting the same measures enforced in 2020.

Nonpharmaceutical interventions have been shown to be effective in preventing the spread of infectious diseases. The strict compliance with nonpharmaceutical interventions implemented during the coronavirus disease (COVID-19) pandemic has been associated with a decline in influenza activity in many countries, including Taiwan (*1*–*4*). Handwashing, disinfecting frequently touched surfaces, and closure of schools might also be effective against nonpolio enteroviruses (NPEV), which commonly cause a spectrum of illnesses in young populations in Asia (*5*). We observed lower NPEV activity during the 2019–20 season in Taiwan compared with the average of the 5 previous seasons, which might be attributable to strict compliance with nonpharmaceutical interventions. We further estimated the protective effect that could have been achieved if the population strictly adhered to the same nonpharmaceutical interventions during those previous seasons.

We collected nationwide data on weekly outpatient and emergency department (ED) visits during November 2014–June 2020 from the Taiwan National Infectious Disease Statistics System (https://nidss.cdc.gov.tw) (*6*). Patients >15 years of age were excluded because of their milder symptoms and low number of cases. The original data were transferred from the National Health Insurance program of Taiwan, which covers >99% of Taiwan residents (Appendix). The Institutional Review Board of the National Health Research Institutes approved this study (approval no. EC1051207-R4).

NPEV activity was measured by using the number of visits that yielded diagnoses of hand, foot, and mouth disease (International Classification of Diseases [ICD], 9th Revision, Clinical Modification, code 074.3 or ICD, 10th Revision, Clinical Modification, code B08.4) or herpangina (ICD, 9th Revision, Clinical Modification, code 074.0 or ICD, 10th Revision, code B08.5). The period from week 47 of 1 year and week 23 of the following year was defined as 1 season. We estimated the change in NPEV activity after the first imported COVID-19 case in Taiwan, when nonpharmaceutical interventions were introduced and enforced, by using a difference-in-difference model used in a previous influenza study (Appendix) (*4*). The total number of outpatient and ED visits for NPEV at baseline was adjusted to eliminate the preintervention differences in NPEV activity between groups (2019–20 season vs. 2014–2019 seasons). The total number of outpatient and ED visits for all disease in different weeks and different years was used for normalization because their numbers decreased after the COVID-19 pandemic. We estimated the preventable fraction among the unexposed (*PF_u_*) to measure the reduction of NPEV that would have been possible in each week of the 2014–2019 seasons, had the same nonpharmaceutical interventions been strictly followed, and adjusted *PF_u_* to control for potential confounder (Appendix).

The number of NPEV visits during the 2019–20 season was 81,942, compared with the average of 205,979 during the 2014–2019 seasons (Appendix Table 1). NPEV activity increased after week 16 across the past 6 seasons except 2019–20, when the earlier low level of weekly activity continued (Figure; Appendix Figure 1). The difference-in-difference analysis revealed that after normalization by visits for NPEV at baseline and for all diseases, NPEV activity during weeks 16–23 in the 2019–20 season was significantly lower than during the same calendar weeks of the 2014–2019 seasons (Appendix Table 2). The lower activity during weeks 16–23 in 2019–20 remained significant across all age groups and hospital settings (Appendix Table 3, 4). The weekly *PF_u_* increased from 73% to 90% (from 17% to 71% for adjusted *PF_u_*) during weeks 16–23 (Table; Appendix Table 5). Similar benefits of the nonpharmaceutical interventions were observed across different age groups of patients and hospital settings (Table; Appendix Table 6).

**Figure:**
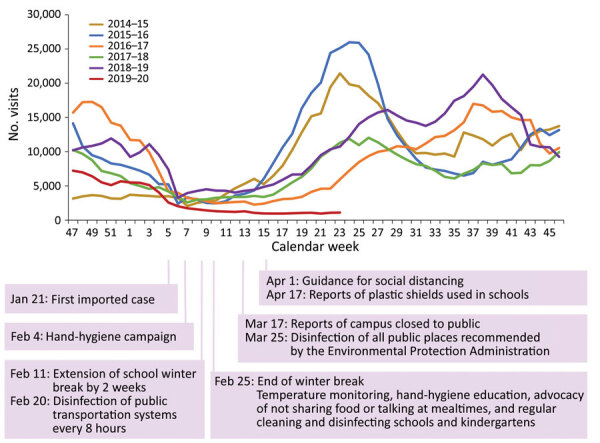
Nonpolio enterovirus activity during 2019–20 season compared with the same weeks in the previous 5 seasons in patients <15 years of age, Taiwan. The y-axis represents the number of outpatient department and emergency department visits in which a diagnosis of hand, foot and mouth disease or herpangina was made for patients <15 years of age.

**Table:** Weekly estimated *PF_u_* during calendar weeks 16–23 in 2020 compared with the same weeks in the previous 5 seasons in patients <15 years of age, by age group, Taiwan*

Calendar week	Estimated *PF_u_* (95% CI)				
	Overall	0–2 y	3–4 y	5–9 y	10–14 y
16	0.73 (0.67–0.78)	0.55 (0.46–0.63)	0.76 (0.70–0.81)	0.78 (0.73–0.83)	0.74 (0.68–0.79)
17	0.76 (0.72–0.80)	0.61 (0.54–0.67)	0.79 (0.75–0.82)	0.81 (0.77–0.85)	0.78 (0.73–0.81)
18	0.79 (0.76–0.82)	0.66 (0.61–0.70)	0.82 (0.79–0.84)	0.84 (0.81–0.86)	0.81 (0.78–0.83)
19	0.82 (0.80–0.84)	0.70 (0.67–0.73)	0.84 (0.82–0.86)	0.86 (0.84–0.88)	0.83 (0.81–0.85)
20	0.84 (0.82–0.86)	0.74 (0.71–0.76)	0.86 (0.84–0.88)	0.88 (0.86–0.89)	0.86 (0.84–0.87)
21	0.86 (0.84–0.88)	0.77 (0.74–0.80)	0.88 (0.86–0.89)	0.89 (0.88–0.91)	0.87 (0.86–0.89)
22	0.88 (0.86–0.90)	0.80 (0.77–0.83)	0.89 (0.87–0.91)	0.91 (0.89–0.92)	0.89 (0.87–0.91)
23	0.90 (0.87–0.91)	0.82 (0.79–0.85)	0.91 (0.89–0.93)	0.92 (0.90–0.94)	0.91 (0.88–0.92)

*All values are statistically significant. *PF_u_*, estimated preventable fraction among the unexposed.

We observed a significant and persistent decrease of NPEV during the 2019–20 season, which might be attributable to strict compliance with the nonpharmaceutical interventions. Up to 90% (71% adjusted) of NPEV activity might have been prevented during the 2014–2019 seasons by adopting the same nonpharmaceutical interventions enforced in 2020. Many factors, such as detection bias and healthcare avoidance, might confound our analyses. However, the detection of NPEV is based on symptoms and was less likely to be affected by the COVID-19 pandemic. In addition, COVID-19 had little impact on the surveillance system in Taiwan because <450 total COVID-19 cases had been reported as of June 17 and no local cases have been reported since April 12. 

Our study is limited by the healthcare avoidance caused by the COVID-19 pandemic (*4*). The normalization procedure using the number of visits for all diseases in our study and subgroup analyses on ED patients (Appendix Table 4, 6) are insufficient to eliminate the impact of healthcare avoidance; active surveillance is required. The effect of individual nonpharmaceutical intervention is difficult to assess. The prolonged winter break might have played a major role in reducing NPEV activity. However, considering the high contagiousness of NPEV, their activity was expected to peak after school reopening if no other interventions were implemented. The persistent low NPEV activity throughout the semester, which began in March 2020, indicated the effectiveness of other interventions.

Appendix Additional information about nonpolio enterovirus activity during the COVID-19 pandemic, Taiwan, 2020.

## References

[1] Kuo SC, Shih SM, Chien LH, Hsiung CA. Collateral benefit of COVID-19 control measures on influenza activity, Taiwan. Emerg Infect Dis. 2020;26:1928–30. 10.3201/eid2608.201192 32339091 PMC7392415

[2] Galvin CJ, Li YJ, Malwade S, Syed-Abdul S. COVID-19 preventive measures showing an unintended decline in infectious diseases in Taiwan. Int J Infect Dis. 2020;98:18–20. 10.1016/j.ijid.2020.06.062 32585283 PMC7308751

[3] Choe YJ, Lee JK. The impact of social distancing on the transmission of influenza virus, South Korea, 2020. Osong Public Health Res Perspect. 2020;11:91–2. 10.24171/j.phrp.2020.11.3.07 32494566 PMC7258883

[4] Sakamoto H, Ishikane M, Ueda P. Seasonal influenza activity during the SARS-CoV-2 outbreak in Japan. JAMA. 2020;323:1969–71. 10.1001/jama.2020.6173 32275293 PMC7149351

[5] Owino CO, Chu JJH. Recent advances on the role of host factors during non-poliovirus enteroviral infections. J Biomed Sci. 2019;26:47. 10.1186/s12929-019-0540-y 31215493 PMC6582496

[6] Jian SW, Chen CM, Lee CY, Liu DP. Real-time surveillance of infectious diseases: Taiwan’s experience. Health Secur. 2017;15:144–53. 10.1089/hs.2016.0107 28418738 PMC5404256

