# Supplementary material for: Nonpolio Enterovirus Activity during the COVID-19 Pandemic, Taiwan, 2020
Source: Emerg Infect Dis. 2021 Jan;27(1):306–8. doi: 10.3201/eid2701.203394 (PMC7774568; doi:10.3201/eid2701.203394)
Supplement: Appendix — Additional information about nonpolio enterovirus activity during the COVID-19 pandemic, Taiwan, 2020. [file 20-3394-Techapp-s1.pdf]

Article DOI: https://doi.org/10.3201/eid2701.203394

Nonpolio Enterovirus Activity during the COVID-19 Pandemic, Taiwan, 2020

Appendix

Methods

Data collection

National Health Insurance provides medical coverage for >99% of Taiwan citizens. It collects medical information of all beneficiaries including ICD-9-CM/ICD-10 codes. Aggregated data regarding outpatient and emergency room (ER) visits are retrieved from National Health Insurance and transferred to Taiwan CDC every week. These data are made public in the Taiwan National Infectious Disease Statistics System (https://nidss.cdc.gov.tw) (*1*). The ICD-9-CM/ICD-10 codes are used to identify hand-foot-and-mouth disease or herpangina (ICD-9-CM of 074.3 or 074.0, and ICD-10 of B08.4 or B08.5 respectively). The diagnoses of hand-foot-and-mouth disease or herpangina are mainly based on symptoms in Taiwan and the ICD-9-CM/ICD-10 codes of beneficiaries are transferred mandatorily to National Health Insurance.

Difference-in-differences model

A “difference-in-differences” model generates a causal estimate of the change in an outcome due to an intervention or exposure, after subtracting the expected background change observed in a reference group without the intervention or exposure. The strengths of this method include preservation of time order, accounting for changes in secular trends, and eliminating the pre-intervention differences in outcomes between the groups. Therefore, this model was used to

analyze data from outpatient department and ER visits for non-polio enterovirus (NPEV) infections. We assigned the whole population of outpatient department and ER visits during the 2019–2020 season to the "exposed" group. In this case, the "exposed" condition means that the population adhered to infection control measures after the first imported COVID-19 case (Figure 1). The populations of outpatient department and ER visits during the past 5 seasons were assigned to five "unexposed" groups. Since the National Health Insurance of Taiwan includes >99% of Taiwan residents, the risk of NPEV is calculated as the ratio of total weekly number of outpatient department and ER visits that yielded an NPEV diagnosis to the total weekly number of outpatient department and ER visits for all diseases.

The basic equation for the model is

$$\mu_{ij} = \beta_0 + \beta_{exp}\text{*Exposure} + \beta_{week} * \text{Week} + \beta_{interaction}\text{*Exposure*Week} + \varepsilon_{ij} \quad (1)$$

where μ_{ij} is the expected mean value for subject *i* at week *j*; Exposure is a binary indicator that the subject is exposed to the infection control measures; Week denotes the specific week; and ε_{ij} is the error term for the outcome measure of subject *i* at week *j*. Note that the outcome measure in this study is the total number of outpatient department and ER visits for NPEV, which is a counting variable. Therefore, in this case the μ_{ij} term on the left-hand side of the equation in Eq. (1) was replaced with $\log(\mu_{ij})$, and the equation is estimated via a Poisson regression model. Thus, the log link is specified and the coefficient estimates are on the log scale. Difference-in-difference values for the 2019–2020 season with respect to the 2014–2019 seasons are presented with a 95% CI. Negative values mean that fewer outpatient department and ER visits for NPEV were observed in the 2019–2020 season than in the 2014 to 2019 seasons.

Poisson regression model

The Poisson regression model was used to estimate the number of outpatient department and ER visits for NPEV per week and to analyze the weekly change in the number of visits for NPEV. The Poisson model is one of the generalized linear models (GLMs) that can deal with strictly positive response variables, such as counts and rates. We assumed that the weekly number of outpatient department and ER visits for NPEV follows a Poisson distribution. That is,

$$y_{ij} \sim \text{Poisson}(\mu_{ij}),$$

where y_{ij} represents the number of outpatient department and ER visits for NPEV in year *i* and week *j*, and μ_{ij} is the expected number of outpatient department and ER visits for NPEV. Our full model, which includes additional interaction terms with sub-periods of the season, is written below. W_{s_t} is a dummy variable for a range of weeks, defined as W_{s_t} = 1 if $s \leq W \leq t$; W_{s_t} = 0, otherwise. Y and W are dummy variables for the year and week; that is, they take the value 1 if the year or week is identical with the index i or j respectively.

$$\begin{aligned}\log(\mu_{ij}) = \log(N_{ij}) &+ \beta_0 + \beta_1 \mathrm{Y} + \beta_2 \mathrm{W} + \beta_3(\mathrm{W}_{1\_7})_{ij} + \beta_4(\mathrm{W}_{8\_11})_{ij} \\ &+ \beta_5(\mathrm{W}_{12\_15})_{ij} + \beta_6(\mathrm{W}_{16\_23})_{ij} + \beta_7(\mathrm{Y} * \mathrm{W})_{ij} + \beta_8(\mathrm{Y} * \mathrm{W}_{1\_7})_{ij} \\ &+ \beta_9(\mathrm{Y} * \mathrm{W}_{8\_11})_{ij} + \beta_{10}(\mathrm{Y} * \mathrm{W}_{12\_15})_{ij} + \beta_{11}(\mathrm{Y} * \mathrm{W}_{16\_23})_{ij} + \beta_{12}(\mathrm{W} * \mathrm{W}_{1\_7})_{ij} \\ &+ \beta_{13}(\mathrm{W} * \mathrm{W}_{8\_11})_{ij} + \beta_{14}(\mathrm{W} * \mathrm{W}_{12\_15})_{ij} + \beta_{15}(\mathrm{W} * \mathrm{W}_{16\_23})_{ij} \\ &+ \beta_{16}(\mathrm{Y} * \mathrm{W} * \mathrm{W}_{1\_7})_{ij} + \beta_{17}(\mathrm{Y} * \mathrm{W} * \mathrm{W}_{8\_11})_{ij} + \beta_{18}(\mathrm{Y} * \mathrm{W} * \mathrm{W}_{12\_15})_{ij} \\ &+ \beta_{19}(\mathrm{Y} * \mathrm{W} * \mathrm{W}_{16\_23})_{ij}\end{aligned}$$

This model has an equivalent representation as follows.

$$\log\left({}^{\mu_{ij}}/_{N_{ij}}\right) = \beta_0 + \beta_1 \mathrm{Y} + \beta_2 \mathrm{W} + \beta_3 (\mathrm{W}_{1\_7})_{ij} + \beta_4 (\mathrm{W}_{8\_11})_{ij}$$
$$+ \beta_5 (\mathrm{W}_{12\_15})_{ij} + \beta_6 (\mathrm{W}_{16\_23})_{ij} + \beta_7 (\mathrm{Y} * \mathrm{W})_{ij} + \beta_8 \left(\mathrm{Y} * \mathrm{W}_{1\_7}\right)_{ij}$$
$$+ \beta_9 (\mathrm{Y} * \mathrm{W}_{8\_11})_{ij} + \beta_{10} (\mathrm{Y} * \mathrm{W}_{12\_15})_{ij} + \beta_{11} (\mathrm{Y} * \mathrm{W}_{16\_23})_{ij} + \beta_{12} (\mathrm{W} * \mathrm{W}_{1\_7})_{ij}$$
$$+ \beta_{13} (\mathrm{W} * \mathrm{W}_{8\_11})_{ij} + \beta_{14} (\mathrm{W} * \mathrm{W}_{12\_15})_{ij} + \beta_{15} (\mathrm{W} * \mathrm{W}_{16\_23})_{ij}$$
$$+ \beta_{16} (\mathrm{Y} * \mathrm{W} * \mathrm{W}_{1\_7})_{ij} + \beta_{17} (\mathrm{Y} * \mathrm{W} * \mathrm{W}_{8\_11})_{ij} + \beta_{18} (\mathrm{Y} * \mathrm{W} * \mathrm{W}_{12\_15})_{ij}$$
$$+ \beta_{19} (\mathrm{Y} * \mathrm{W} * \mathrm{W}_{16\_23})_{ij}$$

where N_{ij} is the weekly total number of outpatient department and ER visits for all diseases in year *i* and week *j*. $\log(N_{ij})$ acts as an offset in the model, which is used to account for the variation in the total number of outpatient department and ER visits for all diseases in different weeks and different years. The parameter β_0 is the intercept; β_1 to β_{19} are unknown parameters to be estimated. With the log link function, μ_{ij} is expressed as a linear function of the explanatory variables. All of the statistical analyses were performed using SAS software, Version 9.4 (SAS Institute Inc., Cary, NC, USA). All *p* values are two-sided and $p < 0.05$ was considered to be statistically significant.

Preventable Fraction among the unexposed

Preventable fraction among the unexposed (PF_u) is the proportion of incidents in the unexposed group that could have been prevented by exposure to infection control measures. It is calculated as

$$PF_u = 1 - RR \quad (2)$$

where *RR* is the relative risk. Risk for NPEV infections among the exposed is defined as the number of weekly outpatient department and ER visits for NPEV out of all individuals seeking health care in the 2019–2020 season. Risk for NPEV infections among the unexposed is

defined as the average number of weekly outpatient department and ER visits for NPEV out of the average number of individuals seeking health care in the 2014–2019 seasons. *RR* is the ratio of the risk in the exposed group to the risk in the unexposed group. The adjusted *RR* is a relative risk adjusted for the total number of outpatient department and ER visits for non-polio enteroviruses and for all diseases, respectively, at week 1. Poisson regression model was used to estimate the adjusted *RR* and the 95% confidence interval. Consequently, adjusted PF_u is obtained.

We also calculate the relative risk and preventable fraction among the unexposed in four age groups (0–2, 3–4, 5–9, and 10–14). These values are denoted by RR_i and $PF_{u,i}$ for an age group i. The PF_u defined in Eq. (2) for patients under age 15 is simply called overall PF_u.

Reference

1. Jian SW, Chen CM, Lee CY, Liu DP. Real-time surveillance of infectious diseases: Taiwan's experience. Health Secur. 2017;15:144–53. PubMed https://doi.org/10.1089/hs.2016.0107

Appendix Table 1. The number of visits for non-polio enteroviruses during the 2019–2020 versus 2014–2019 seasons for patients under age 15.

	Number of visits (Week 47-Week 23)	
Characteristic	2019–2020	2014–2019 (Average)
Overall	81,942	205,979
Age group, y		
0–2	26,439	61,255
3–4	24,039	62,541
5–9	26,257	68,403
10–14	5,207	13,780
Hospital setting		
Emergency room	3,784	10,018
Outpatient department	78,158	195,961

Appendix Table 2. Difference in the estimated number of outpatient department and emergency room (ER) visits for non-polio enteroviruses (NPEV) during the 2019–2020 season compared to the 2014–2019 seasons for patients under age 15

	Estimated No. of visits		Difference-in-difference value in 2019–2020 vs
Calendar week	2019–2020 season	2014–2019 seasons	2014–2019 seasons (95% CI)[a,b,c]
47	7384	10756	
48	6502	10308	
49	6015	9876	
50	6456	9650	
51	5613	8879	
52	4985	8481	
1	5844	8337	1006 (514, 1542)
2	5177	7004	1672 (1336, 2032)
3	4802	6517	1784 (1522, 2062)
4	4236	5941	1793 (1544, 2058)
5	2511	5112	898 (710, 1101)
6	2308	3352	2456 (2233, 2702)
7	1644	3174	1969 (1772, 2194)
8	1628	3081	2046 (1759, 2394)
9	1346	3202	1643 (1480, 1829)

Calendar week	Estimated No. of visits 2019–2020 season	Estimated No. of visits 2014–2019 seasons	Difference-in-difference value in 2019–2020 vs 2014–2019 seasons (95% CI)[a,b,c]
10	1337	3178	1658 (1487, 1856)
11	1269	3263	1506 (1265, 1803)
12	1324	3604	1219 (965, 1533)
13	1186	4045	640 (483, 822)
14	996	4077	419 (278, 582)
15	1077	4389	187 (−36, 468)
16	1092	5582	**−991 (−1172, −773)**
17	979	6648	**−2170 (−2305, −2013)**
18	949	7656	**−3208 (−3315, −3086)**
19	974	8752	**−4278 (−4374, −4173)**
20	1008	10213	**−5706 (−5803, −5599)**
21	957	11852	**−7396 (−7501, −7278)**
22	1089	13451	**−8863 (−9008, −8696)**
23	1226	15612	**−10886 (−11084, −10651)**

[a]Subtraction of differences before week 1 from week 1 to 23 differences. Negative values represented fewer NPEV outpatient department and ER visits in the 2019–2020 season vs the 2014–2019 seasons.

[b]Normalized by the total number of outpatient department and ER visits for NPEV at baseline (weeks 47–52) to eliminate the pre-intervention differences in outcomes between the groups (the 2019–2020 season versus the average of five previous seasons). The variation in the total number of outpatient department and ER visits for all diseases in different weeks and different years had been adjusted through the offset in the model.

[c]The decrease with statistical significance was indicated by bold type.

Appendix Table 3. Difference in the estimated number of outpatient department and emergency room (ER) visits for non-polio enteroviruses (NPEV) during 2019–2020 compared to the 2014–2019 seasons in different age groups

Calendar	Difference-in-difference value in 2019–2020 vs 2014–2019 seasons (95% CI)[a,b,c]			
week	Age 0–2	Age 3–4	Age 5–9	Age 10–14
1	925 (773, 1094)	331 (173, 503)	**−226 (−428, −4)**	−27 (−63, 13)
2	865 (772, 966)	499 (394, 612)	188 (44, 343)	49 (23, 76)
3	968 (894, 1048)	581 (499, 669)	198 (86, 317)	56 (36, 77)
4	945 (879, 1016)	590 (512, 673)	256 (147, 373)	58 (39, 78)
5	798 (739, 863)	291 (228, 358)	**−128 (−203, −46)**	17 (3, 32)
6	1130 (1054, 1215)	682 (612, 758)	466 (382, 560)	150 (132, 170)
7	935 (865, 1015)	522 (462, 590)	341 (269, 426)	120 (103, 138)
8	999 (902, 1118)	597 (511, 704)	327 (222, 461)	92 (69, 121)
9	937 (880, 1003)	508 (457, 568)	185 (123, 257)	51 (39, 65)
10	970 (909, 1040)	466 (414, 527)	174 (109, 251)	64 (51, 79)
11	945 (860, 1050)	373 (306, 459)	95 (4, 211)	70 (51, 94)
12	958 (862, 1077)	292 (220, 382)	−68 (−159, 51)	43 (24, 69)
13	742 (685, 807)	139 (94, 192)	**−251 (−309, −182)**	−4 (−16, 10)
14	644 (593, 705)	100 (57, 151)	**−286 (−338, −223)**	**−33 (−42, −21)**
15	512 (433, 612)	58 (−9, 143)	**−323 (−405, −215)**	**−61 (−76, −40)**
16	287 (220, 368)	**−352 (−402, −291)**	**−830 (−896, −747)**	**−98 (−112, −81)**
17	−13 (−64, 46)	**−696 (−734, −650)**	**−1270 (−1320, −1211)**	**−184 (−194, −172)**
18	**−306 (−347, −260)**	**−1007 (−1038, −972)**	**−1642 (−1682, −1596)**	**−259 (−267, −250)**
19	**−614 (−650, −574)**	**−1319 (−1345, −1290)**	**−2014 (−2049, −1974)**	**−342 (−350, −334)**
20	**−956 (−993, −916)**	**−1747 (−1774, −1718)**	**−2551 (−2587, −2511)**	**−455 (−463, −447)**
21	**−1374 (−1415, −1328)**	**−2224 (−2254, −2191)**	**−3159 (−3198, −3115)**	**−604 (−612, −595)**
22	**−1758 (−1812, −1696)**	**−2706 (−2746, −2659)**	**−3693 (−3747, −3628)**	**−718 (−729, −705)**
23	**−2252 (−2323, −2167)**	**−3297 (−3351, −3231)**	**−4451 (−4525, −4359)**	**−893 (−909, −874)**

[a]Subtraction of differences before week 1 from week 1 to 23 differences. Negative values represented fewer NPEV outpatient department and ER visits during the 2019–2020 season vs the 2014–2019 seasons.

[b]Normalized by the total number of outpatient department and ER visits for NPEV at baseline (weeks 47–52) to eliminate the pre-intervention differences in outcomes between the groups (the 2019–2020 season versus the average of five previous seasons). The variation in the total number of outpatient department and ER visits for all diseases in different weeks and different years had been adjusted through the offset in the model.

[c]The decrease with statistical significance was indicated by bold type.

Appendix Table 4. Difference in the estimated number of outpatient department or emergency room (ER) visits for non-polio enteroviruses (NPEV) during the 2019–2020 season compared to the 2014–2019 seasons for patients under age 15

	Difference-in-difference value in 2019–2020 vs 2014–2019 seasons (95% CI)[a,b,c]	
Calendar week	Outpatient department	Emergency room
1	896 (414, 1423)	78 (50, 110)
2	1553 (1224, 1905)	108 (88, 129)
3	1683 (1426, 1956)	92 (79, 107)
4	1669 (1427, 1927)	178 (159, 198)
5	825 (645, 1020)	164 (144, 186)
6	2419 (2201, 2660)	**−31 (−42, −18)**
7	1942 (1750, 2161)	0 (−11, 13)
8	1964 (1684, 2307)	73 (57, 93)
9	1559 (1400, 1740)	98 (86, 112)
10	1581 (1412, 1775)	77 (68, 88)
11	1435 (1198, 1730)	67 (55, 83)
12	1175 (925, 1486)	48 (35, 64)
13	631 (476, 811)	8 (0, 17)
14	432 (295, 593)	**−21 (−31, −9)**
15	210 (−10, 488)	**−18 (−28, −4)**
16	**−939 (−1116, −725)**	**−57 (−68, −44)**
17	**−2062 (−2195, −1908)**	**−112 (−119, −104)**
18	**−3046 (−3152, −2926)**	**−159 (−165, −152)**
19	**−4070 (−4163, −3966)**	**−208 (−214, −202)**
20	**−5434 (−5529, −5328)**	**−266 (−272, −259)**
21	**−7049 (−7152, −6933)**	**−336 (−341, −329)**
22	**−8419 (−8561, −8255)**	**−443 (−452, −432)**
23	**−10317 (−10513, −10083)**	**−555 (−567, −541)**

[a]Subtraction of differences before week 1 from week 1 to 23 differences. Negative values represented fewer NPEV outpatient department or ER visits in the 2019–2020 season vs the 2014–2019 seasons.

[b]Normalized by the total number of outpatient department or ER visits for NPEV at baseline (weeks 47–52) to eliminate the pre-intervention differences in outcomes between the groups (the 2019–2020 season versus the average of five previous seasons). The variation in the total number of outpatient department or ER visits for all diseases in different weeks and different years had been adjusted through the offset in the model.

[c]The decrease with statistical significance was indicated by bold type.

Appendix Table 5. Weekly Estimated Preventable Fraction among the unexposed (PF_u) from week 16-week 23 of year 2020, compared to the same weeks in the previous five seasons[a] in different age groups. The PF_u here was adjusted by total number of outpatient department and emergency room visits for non-polio enteroviruses and for all diseases, respectively, at week 1

	Estimated Preventable Fraction among the unexposed (95% CI)[a]				
Calendar week	Overall	Age 0–2	Age 3–4	Age 5–9	Age 10–14
16	0.17 (0.05, 0.28)	0.09 (−0.06, 0.22) [b]	0.14 (0.01, 0.25)	0.13 (−0.01, 0.25) [b]	0.04 (−0.10, 0.16) [b]
17	0.29 (0.20, 0.36)	0.21 (0.10, 0.30)	0.26 (0.16, 0.34)	0.26 (0.16, 0.34)	0.19 (0.09, 0.27)
18	0.38 (0.33, 0.44)	0.31 (0.24, 0.38)	0.36 (0.30, 0.42)	0.36 (0.30, 0.42)	0.31 (0.25, 0.37)
19	0.47 (0.43, 0.51)	0.40 (0.35 0.45)	0.45 (0.40, 0.49)	0.46 (0.41, 0.50)	0.41 (0.37, 0.46)
20	0.54 (0.51, 0.58)	0.48 (0.43, 0.52)	0.52 (0.48, 0.56)	0.53 (0.50, 0.57)	0.50 (0.47, 0.54)
21	0.61 (0.57, 0.64)	0.55 (0.50, 0.59)	0.59 (0.55, 0.62)	0.60 (0.56, 0.64)	0.58 (0.54, 0.61)
22	0.66 (0.62, 0.69)	0.61 (0.56, 0.65)	0.64 (0.60, 0.68)	0.66 (0.62, 0.70)	0.64 (0.61, 0.68)
23	0.71 (0.67, 0.74)	0.66 (0.61, 0.70)	0.69 (0.65, 0.73)	0.71 (0.67, 0.75)	0.70 (0.66, 0.73)

[a]Normalized by the total number of outpatient department and emergency room visits for non-polio enteroviruses and for all diseases, respectively, at week 1.

[b] All values were statistically significant except those underlined.

Appendix Table 6. Weekly Estimated Preventable Fraction among the unexposed (PF_u) from week 16-week 23 of year 2020, compared to the same weeks in the previous five seasons in different hospital settings

	Estimated Preventable Fraction among the unexposed (95% CI)[a]	
Calendar week	Outpatient department	Emergency room
16	0.73 (0.67, 0.78)	0.63 (0.53, 0.71)
17	0.76 (0.72, 0.80)	0.68 (0.61, 0.74)
18	0.79 (0.76, 0.82)	0.72 (0.67, 0.76)
19	0.82 (0.80, 0.84)	0.76 (0.72, 0.79)
20	0.84 (0.83, 0.86)	0.79 (0.76, 0.81)
21	0.86 (0.85, 0.88)	0.81 (0.79, 0.84)
22	0.88 (0.86, 0.90)	0.84 (0.81, 0.87)
23	0.90 (0.87, 0.91)	0.86 (0.82, 0.89)

[a]All values were statistically significant.

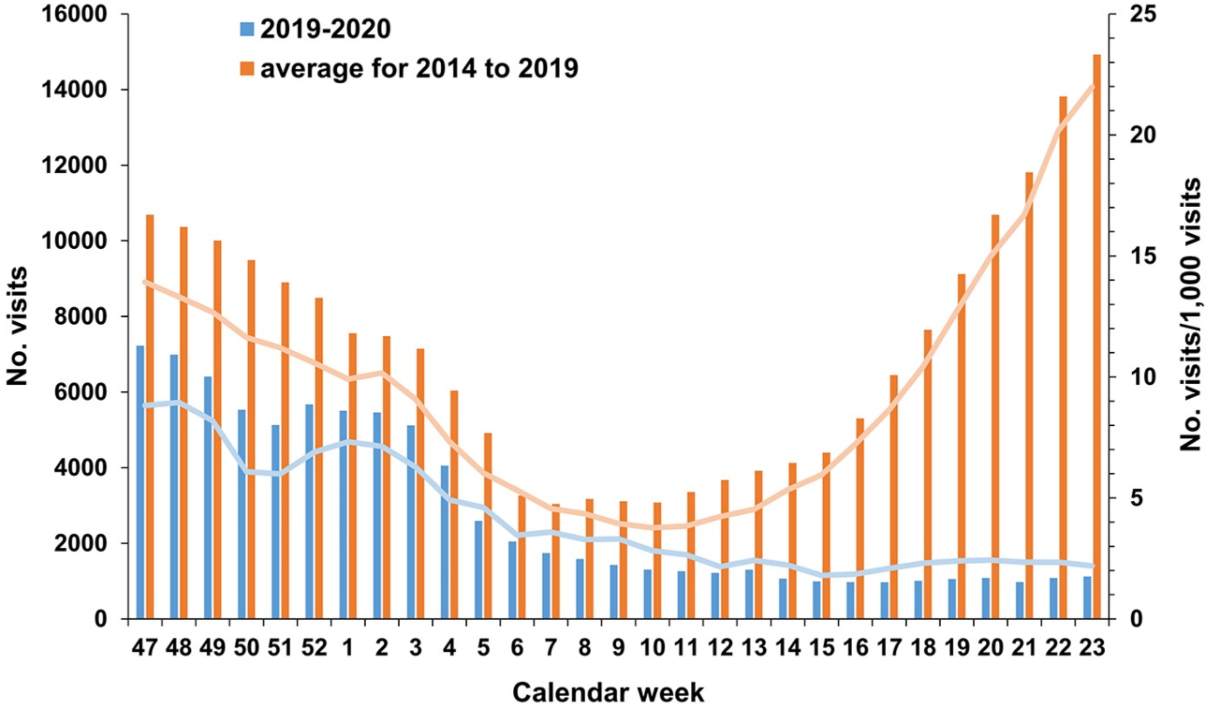

Appendix Figure. Non-polio enterovirus activity in Taiwan during 2019–2020 compared to the average of 2014–2019 for patients under age 15. The line represents the hand-foot-and-mouth disease or herpangina diagnoses per 1,000 visits. The bar represents the number of outpatient department and emergency room visits in which a diagnosis of hand-foot-and-mouth disease or herpangina was made.
